# Nucleoplasmic calcium signaling and cell proliferation: calcium signaling in the nucleus

**DOI:** 10.1186/1478-811X-11-14

**Published:** 2013-02-21

**Authors:** Rodrigo R Resende, Lidia M Andrade, Andre G Oliveira, Erika S Guimarães, Silvia Guatimosim, M Fatima Leite

**Affiliations:** 1 Department of Biochemistry and Immunology, Federal University of Minas Gerais, Belo Horizonte, MG, Brazil; 2 René Rachou Research Center, Oswaldo Cruz Foundation, Belo Horizonte, MG, Brazil; 3 Physiology and Biophysics, Federal University of Minas Gerais, Belo Horizonte, MG, Brazil; 4 Howard Hughes Medical Institute, Chevy Chase, Maryland, MD, USA; 5 Institute of Biological Sciences Bloco A4 246, Federal University of Minas Gerais, Av Antônio Carlos, 6627, Belo Horizonte, 30370-920, Brazil

**Keywords:** Nucleoplasmic reticulum, Nuclear calcium channels, Nuclear calcium, Cell proliferation

## Abstract

Calcium (Ca^2+^) is an essential signal transduction element involved in the regulation of several cellular activities and it is required at various key stages of the cell cycle. Intracellular Ca^2+^ is crucial for the orderly cell cycle progression and plays a vital role in the regulation of cell proliferation. Recently, it was demonstrated by *in vitro* and *in vivo* studies that nucleoplasmic Ca^2+^ regulates cell growth. Even though the mechanism by which nuclear Ca^2+^ regulates cell proliferation is not completely understood, there are reports demonstrating that activation of tyrosine kinase receptors (RTKs) leads to translocation of RTKs to the nucleus to generate localized nuclear Ca^2+^ signaling which are believed to modulate cell proliferation. Moreover, nuclear Ca^2+^ regulates the expression of genes involved in cell growth. This review will describe the nuclear Ca^2+^ signaling machinery and its role in cell proliferation. Additionally, the potential role of nuclear Ca^2+^ as a target in cancer therapy will be discussed.

## Introduction

Intracellular calcium (Ca^2+^) participates as a second messenger in several signaling pathways coordinating key events in a variety of cellular functions [1]. Ca^2+^ Signals are generally initiated by the binding of a hormone or growth factor to a transmembrane receptor, most commonly G protein coupled receptor (GPR) or tyrosine kinase receptor (RTK). The activation of such receptors recruits several second messengers, including phospholipase C (PLC) that, once activated, cleaves phosphatidylinositol 4,5-biphosphate (PIP_2_) producing diacylglycerol and inositol-1,4,5-trisphosphate (InsP_3_). InsP_3_ then binds to the InsP_3_ receptor (InsP_3_R), activating its channel to release Ca^2+^ from the endoplasmic reticulum. Once in the cytosol, Ca^2+^ can participate in several intracellular cascades and activate another class of Ca^2+^ channels, the ryanodine receptor (RyR), triggering a process denoted Ca^2+^-induced Ca^2+^ release [1]. The type II and III RyR are also sensitive to cyclic ADP-ribose (cADPR) [2,3], a process first demonstrated in sea urchin eggs [4], but now known to mobilize Ca^2+^ in a wide range of mammalian cell types [3,5-8]. Members of a third family of intracellular Ca^2+^ channels, the two pore channels (TPCs), are activated by nicotinic acid adenine dinucleotide phosphate (NAAD) which promotes release of Ca^2+^ from acidic organelles [9,10]. Like cADPR, NAADP was discovered in sea urchin eggs [11] and has now been found to induce Ca^2+^ signaling in mammalian cells as well [9,10]. Interactions among these different intracellular Ca^2+^ channels coordinate cellular responses mediated by Ca^2+^, both in health and disease. However little is known regarding the interaction of intracellular Ca^2+^ channels in the regulation of nuclear Ca^2+^ signaling.

One way by which intracellular Ca^2+^ regulates multiple cell functions is through spatial segregation of Ca^2+^ signaling. Indeed, subcellular increases in Ca^2+^ modulate not only physiological but also pathological events. For example, the physiological secretion of zymogen granules in pancreatic acinar cells is triggered by a localized sub-apical Ca^2+^ increase that does not spread throughout the entire cell [12]. In the other hand, the hypertrophic response in cardiomyocytes depends mostly on nuclear Ca^2+^ signals [13]. Additional examples of cellular processes modulated by subcellular Ca^2+^ signaling include the extension of growth cones in neuronal cells [14,15] and the establishment of specific gene transcription signatures [16], regulating development and differentiation [17-20], among others [21].

Moreover, the presence of another regulatory nuclear Ca^2+^ domain, denoted the nucleoplasmic reticulum [22,23] was reported in a wide variety of cells, from plants to animals (reviewed in [24]). The nucleoplasmic reticulum, of which 2 classes have been described, is a reticular membrane network of Ca^2+^ stores that is continuous with the endoplasmic reticulum and the nuclear envelope. The type I contains invaginations of the inner membrane of the nuclear envelope, and the type II contains both the inner and outer nuclear envelope membrane. These two classes of the nucleoplasmic reticulum can coexist within the same nucleus (reviewed in [24]), and their structure undergoes dynamic remodeling [25]. With the capacity to regulate Ca^2+^ signals in subnuclear regions, the presence of such machinery might provide a potential mechanism by which nucleoplasmic Ca^2+^ could simultaneously regulate many independent processes in the nucleus.

**Figure 1:**
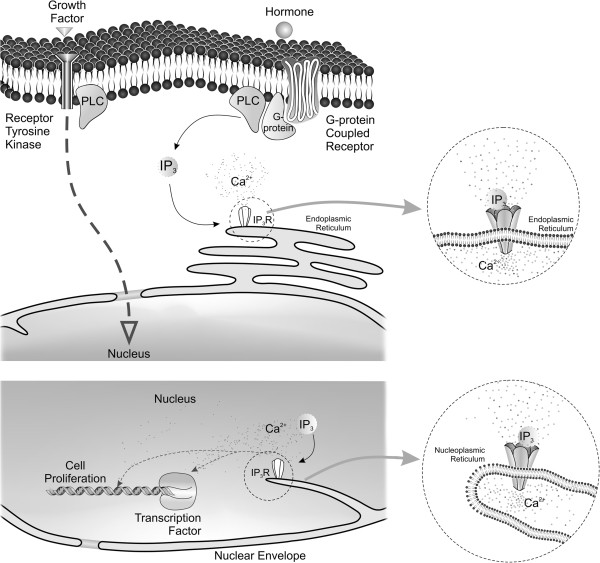
**Schematic representation of events involved in nuclear Ca**^**2+ **^**release and cellular proliferation.** Ca^2+^ signals can be activated in the cells by the binding of a hormone or growth factor to a transmembrane receptor, most commonly a G protein coupled receptor or tyrosine kinase receptor. Through complex signaling cascades and networks, these effectors lead to the activation of several second messengers. One of the signaling pathways activated during cell proliferation is phospholipase C (PLC) that, once activated, cleaves phosphatidylinositol 4,5-biphosphate (PIP_2_) producing diacylglycerol and inositol-1,4,5-trisphosphate (InsP_3_). InsP_3_ then binds to the InsP_3_ receptor (InsP_3_R), activating its channel to release Ca^2+^. InsP_3_Rs can be found in the endoplasmic reticulum, in the nuclear envelope and in the nucleus along the nucleoplasmic reticulum. InsP_3_R-induced Ca^2+^ release specifically in the nucleus has been involved in the regulation of gene expression during different pathophysiological conditions, as well as during cellular proliferation. The cartoon shows the type I nucleoplasmic reticulum structure, with an invagination of the inner nuclear membrane alone. A Type II nucleoplasmic reticulum structure, with a double membrane walled invagination has also been reported in many cell types, although it is not represented here. In the nucleus, these cellular invaginations can provide focal release of Ca^2+^ that can bind directly to DNA structure or can modulate transcription factors involved in cell proliferation.

Although it is well known that nuclear Ca^2+^ has biological effects that differ from those mediated by increases in cytosolic Ca^2+^[21], the mechanisms by which Ca^2+^ is specifically increased in the nucleoplasm are a topic of debate. It was initially proposed that nuclear Ca^2+^ signaling would occur by passive diffusion of cytosolic Ca^2+^ across the nuclear envelope into the nucleoplasm. However, as it will be discussed in more detail, the nuclear interior has all the machinery required to produce localized Ca^2+^ signals, supporting the concept of the nuclear compartment as an independent apparatus to trigger Ca^2+^ signals. Moreover, the mechanisms and pathways by which localized Ca^2+^ signals in the nucleus regulate cell growth have only recently been investigated. The emerging model (Figure 1) shows that upon growth factor stimulation, RTKs translocate to the nucleus to induce hydrolysis of nuclear PIP2, generating InsP3 in the nucleoplasm, which leads to nuclear Ca^2+^ signals that can control cell growth [26-28] (Figure 1). This review highlights recent advances on nuclear Ca^2+^ signaling and its role in cell proliferation.

### Ca^2+^ signaling in the nucleus

The nucleus is separated from the cytosol by the nuclear envelope, which is a specialized region of the endoplasmic reticulum, comprised of phospholipid bilayers [24]. However, the nuclear envelope contains pores that are permeable to molecules up to 60 kDa in size [29]. In the absence of a gating mechanism, a pore of this size would allow rapid equilibration of Ca^2+^ between the nucleus and cytosol. Indeed, under certain circumstances, free diffusion of Ca^2+^ through the nuclear pore occurs [30]. For example, stimulation of basophilic leukemia cells with antigen or photoreleased InsP_3_ triggered Ca^2+^ waves that spread from the cytosol into the nucleus [31]. Similar observations have been made in hepatocytes stimulated with vasopressin [32]. In contrast, several reports have demonstrate the existence of a nuclear-cytosolic Ca^2+^ gradient in a number of cell types [33,34], indicating that the permeability of nuclear pores to this ion can be regulated. However, the detection of such gradient can be incorrectly inferred depending on the technique used to measure intracellular Ca^2+^ in different compartments. Some of the commonly used organic Ca^2+^ indicators can display uneven distribution in the interior of the cells and can preferentially accumulate in membrane compartments such as the ER and the nucleoplasm. More importantly, the affinity of fluorescent probes for Ca^2+^ can vary depending on the cellular environment (reviewed [35]). Although each method for analyzing Ca^2+^ has certain drawbacks it is now appreciated that Ca^2+^ signaling is regulated at the subcellular level, and that this level of regulation is necessary for Ca^2+^ to act as a second messenger that regulates multiple cell functions simultaneously.

The nuclear envelope itself is a Ca^2+^ rich compartment, accumulating Ca^2+^ via a Ca^2+^-ATPase pump (SERCA) and a Na^+^/Ca^2+^-exchanger [36-38] and releasing it via channels that are sensitive to InsP_3_[37,39], cADPR [39,40], and NAADP [41]. The Ca^2+^-ATPase pump was shown to be present only in the outer membrane of the nuclear envelop, while the Na^+^/Ca^2+^-exchanger, is expressed in the inner membrane [38,42]. Regarding the intracellular Ca^2+^ channels, the RyRs appears to be present on both leaflets of the nuclear envelope [43]. Similarly, there are reports of InsP_3_Rs in the inner and outer membrane [28,44,45]. In addition, it was shown that ADP-ribosyl (CD38), an enzyme required for generation of cADPR, is located on the inner membrane of the nuclear envelope [40]. In *Aplysia* neurons, depolarization is the signal that triggers the translocation of CD38 to the nucleus [46]. Moreover, the nuclear envelope possess the tool kit necessary to produce InsP_3_, including PIP2, and PLC [47], and this machinery may be activated selectively through tyrosine kinase pathway [48].

However, the nuclear envelope is not the only nuclear site containing the Ca^2+^ signaling machinery. The nucleoplasmic reticulum represents another specialized cellular compartment involved in regulation in time and space of specific intracellular Ca^2+^ signaling events. For instance, both the InsP_3_R and the RyR are found in the nucleoplasmic reticulum [22,23]. Importantly, the InsP_3_-Kinase (IP3KB), the isoform that inactivates the InsP_3_ by phosphorylating it, was also reported to be located in the nucleoplasmic reticulum, where it may function to terminate the InsP_3_ mediated Ca^2+^ signal [49]. In addition, SERCA was also shown to be expressed along invaginations of the nucleoplasmic reticulum [50]. Therefore, there are several reports describing an active Ca^2+^ signaling regulatory domain deep in the nucleus, along the nucleoplasmic reticulum, providing further spatial control of Ca^2+^ within this cellular compartment [22,51,52].

Corroborating these findings, there is a growing body of data demonstrating that the nucleus has the capacity to independently generate Ca^2+^ signals. Several *in vitro* studies have shown that InsP_3_ releases Ca^2+^ directly from the nuclear envelop into the nucleus [33,39,44,53,54]. Accordingly, it has been demonstrated in a liver cell line that extracellular ATP can activate nuclear Ca^2+^ release, via an InsP_3_-dependent mechanism [55]. In cardiomyocytes, endothelin-1 has also been shown to elicit a local nuclear envelope Ca^2+^ release via InsP_3_R that activates nuclear CaMKII triggering HDAC5 phosphorylation and its nuclear export [56]. This signaling pathway has been implicated in the regulation of gene transcription in adult ventricular myocytes in response to neurohumoral signals during hypertrophy. Similar to InsP_3_, cADPR can also increase Ca^2+^ in isolated cell nuclei [33,39,40].

One of the proposed mechanism by which InsP_3_ generates nuclear Ca^2+^ signaling is via translocation of activated RTKs from the plasma membrane to the nuclear interior. For instance, it was shown that IGF-1 and integrins caused PIP2 breakdown in the nucleus but not at the plasma membrane [48]. Similarly, activation of the hepatocyte growth factor (HGF) receptor c-Met in a liver cell line and insulin receptor in primary hepatocytes caused PIP2 breakdown in the nucleus resulting in InsP_3_ formation that was followed by nuclear Ca^2+^ signals [26,27] (Figure 1). The triggering of this highly localized cascade was dependent on the rapid translocation of the activated HGF receptor to the nucleus, through a mechanism that depends on the adaptor protein Gab-1 and importin-B [26]. Moreover, it also has been hypothesized that relocation of MAP kinase to the nucleus activates nuclear phospholipase C to generate InsP_3_ there [43].

Once in the nucleus, Ca^2+^ signals directly regulate signaling pathways distinct from those mediated by cytosolic Ca^2+^, for instance they stimulate the intranuclear activity of PKC [22] and CaMK-IV [57]. Nuclear Ca^2+^ also plays a significant role in regulating the transcription factor CRE-binding protein and its coactivator, CREB-binding protein (CBP) [58]. Transcriptional activation of Elk-1 by EGF was also shown to depend on nuclear rather than cytosolic Ca^2+^[59]. On the other hand, nuclear Ca^2+^ can negatively regulate the activity of transcription factors, such as TEAD [60]. Moreover, nuclear Ca^2+^ has also been implicated in modulating cardiac hypertrophy [13,51] and within the nucleus Ca^2+^ was shown to bind to and directly regulate DNA structure [61]. Another evidence of the role of nuclear Ca^2+^ signaling pathway came from studies showing that in skeletal muscle cell, two-photon photorelease of caged Ca^2+^ near the nucleoplasmic reticulum was found to elicit a Ca^2+^-induced Ca^2+^ release event within the nucleus [23]. More recently, it was demonstrated that nuclear rather than cytosolic Ca^2+^ signals specifically control the progression through early prophase, showing that nucleoplasmic Ca^2+^ regulates cell proliferation [62].

### Nuclear Ca^2+^ and cell proliferation

It has been long recognized that Ca^2+^ signals have an important role throughout the mammalian cell cycle and are especially important in early G_1_ and G_1_/S and G_2_/M transitions [63], with the first major Ca^2+^ transient occurring just prior to entry into mitosis, and the second one occurring during the metaphase-anaphase transition [63,64]. Indeed, Ca^2+^ is the most prominent messenger required through these cycle points [65,66] and downstream targets of Ca^2+^ have also been implicated in cell cycle progression as well [67,68].

Heterologous expression of the Ca^2+^ binding protein parvalbumin has been used to study the role of Ca^2+^ signals in the regulation of the cell cycle. This protein is normally expressed in skeletal muscle and neurons [69], and is known to buffer Ca^2+^[70]. The first report using parvalbumin as a molecular tool to buffer intracellular Ca^2+^ and study cellular growth, showed that reducing Ca^2+^ slowed progression through the cell cycle [68]. However, it is now known that the effects of Ca^2+^ on proliferation correlate with the subcellular compartment where Ca^2+^ is released. Using parvalbumin variants, selectively targeted to distinct intracellular Ca^2+^ rich compartments, it was found that buffering mitochondrial Ca^2+^ inhibits apoptosis and accelerates hepatocyte proliferation [71]. In contrast, buffering cytosolic Ca^2+^ was shown to retard liver regeneration and progression through the cell cycle after partial hepatectomy [72]. Since cytosolic Ca^2+^ can increase through a number of mechanisms, it is believed that, in this compartment, Ca^2+^ may have different effects on cell growth [71,73,74]. On the other hand, it was shown that nucleoplasmic rather than cytosolic Ca^2+^ is essential for liver cell line proliferation, and is necessary in particular for progression through early prophase [62]. It was also found that liver tumors implanted in nude mice grew much more slowly when expressing parvalbumin in their nuclei compared to the cytosol [62]. Moreover, HGF and insulin, two potent growth factors in liver, that induce cell proliferation during liver regeneration, were shown to selectively form InsP_3_ in the nucleus to initiate nuclear Ca^2+^ signals [22,27] (Figure 1). Since the nucleoplasmic reticulum is known to be abundant in many tumor cell types [24], one would expect that the existence of these nuclear invaginations could provide further specificity to cell proliferation by allowing the focal delivery of Ca^2+^ to particular sites within the nucleus.

Although, the proteins that link nuclear Ca^2+^ signals to cell proliferation have not been clearly identified, more recent findings in liver tumor cells indicated the endopeptidase legumain (LGMN) as a novel target of nuclear Ca^2+^[75]. Using Rapid Subtraction Hybridization (RaSH) to subtract genes in liver cells expressing the Ca^2+^ buffer protein parvalbumin targeted to the nucleus, from genes in cells expressing a mutated form of nuclear-targeted parvalbumin which has one of the two Ca^2+^-binding sites inactivated. The authors identified thirteen genes whose expression was affected by a small alteration in nuclear Ca^2+^ concentration. LGMN was one of such genes and upon further validation was demonstrated to be regulated by nuclear Ca^2+^ signals at the transcriptional level. LGMN was first recognized in plants [76] and later in humans and mice [77]. It is present in the tumor microenvironment where it is expressed by macrophages and contributes to metastatic behavior by promoting cell migration and tissue invasion. It is known that increased expression of LGMN is associated with poor tumor differentiation [78]. For instance, it was demonstrated that LGMN co-localizes with integrins at the invading front of tumors and expression of this enzyme was shown to be associated with increased invasiveness [78,79]. So, it was shown that when Ca^2+^ was buffered in the nucleus of the cells, LGMN expression decreased, impairing cell proliferation [75]. Additionally, this work also provided evidence that nuclear Ca^2+^ signals regulate cell proliferation at least in part through the modulation of gene expression (Figure 1). Other targets for nuclear Ca^2+^ that are involved in cell proliferation still remain to be described.

Altered nuclear morphology is a common feature of many cancers [24] and it has been proposed that information regarding the nucleoplasmic reticulum invaginations could be used in combination with other nuclear anomalies as markers of malignancy [80]. More recently, it was also proposed that nuclear Ca^2+^ buffering could be used in conjunction with radiotherapy as a therapeutic potential for the treatment of carcinoma. Ionizing radiation concomitant with nuclear Ca^2+^ buffering showed superior outcome, compared to irradiation alone [81]. Corroborating previous findings, the beneficial effect of nuclear Ca^2+^ buffering in the proposed antitumor therapy was shown to be due to changes caused in expression level of genes involved in the regulation of cell proliferation [59]. Moreover, it was also shown that buffering nuclear Ca^2+^ reduced the rate of tumor cell proliferation, without affecting cells from normal tissue [81], suggesting higher selectivity of nuclear Ca^2+^ towards controlling cancer cell growth. Further studies are required to determine the mechanistic basis for the differential sensitivity of normal versus cancer cell proliferation to changes in nuclear Ca^2+^. Nonetheless, these findings suggest that buffering nuclear Ca^2+^ could be one strategy employed to inhibit the growth of tumors without affecting normal tissue, either alone or in association therapy.

## Conclusions

Ca^2+^ is important to several signaling pathways among virtually every cell type. The central mechanism by which Ca^2+^ regulates protein functions depends on how and where it is released into the cell. The role of nuclear Ca^2+^ in cell proliferation was demonstrated *in vitro* by showing that nuclear Ca^2+^ buffering reduced proliferation rate through blocking cell cycle in G_2_/M phase. It was also demonstrated that nuclear Ca^2+^ plays a role on tumor growth *in vivo* and it can alter the expression of genes involved in cell proliferation. Moreover, modulation of nuclear Ca^2+^ signaling was shown to be a potential target to treat cancer. However further studies are needed to better understand how nuclear Ca^2+^ can be generated and how it regulates cell proliferation and cell cycle progression. These findings would have strong potential as therapeutic targets in degenerative diseases or cancer.

## Competing interests

The authors declare that they have no competing interests.

## Authors’ contributions

All authors contributed in the conception and writing of the manuscript. All authors edited and approved the final version.
